# Effects of point and nonpoint source controls on total phosphorus load trends across the Chesapeake Bay watershed, USA

**DOI:** 10.1088/1748-9326/ad0d3c

**Published:** 2023-12-01

**Authors:** Qian Zhang, Joel T Bostic, Robert D Sabo

**Affiliations:** 1 University of Maryland Center for Environmental Science, Annapolis, MD, United States of America; 2 University of Maryland Center for Environmental Science, Frostburg, MD, United States of America; 3 Garrett College, McHenry, MD, United States of America; 4 U.S. Environmental Protection Agency, Office of Research and Development, Washington, DC, United States of America

**Keywords:** water quality, point source, nonpoint source, watershed management, cluster analysis, random forest

## Abstract

Reduction of total phosphorus (TP) loads has long been a management focus of Chesapeake Bay restoration, but riverine monitoring stations have shown mixed temporal trends. To better understand the regional patterns and drivers of TP trends across the Bay watershed, we compiled and analyzed TP load data from 90 non-tidal network stations using clustering and random forest (RF) approaches. These stations were categorized into two distinct clusters of short-term (2013–2020) TP load trends, i.e. monotonic increase (*n* = 35) and monotonic decline (*n* = 55). RF models were developed to identify likely regional drivers of TP trend clusters. Reductions in point sources and agricultural nonpoint sources (i.e. fertilizer) both contributed to water-quality improvement in our period of analysis, thereby demonstrating the effectiveness of nutrient management and the importance of continuing such efforts. In addition, declining TP trends have a larger chance to occur in carbonate areas but a smaller chance in Coastal Plain areas, with the latter likely reflecting the effect of legacy P. To provide spatially explicit information, TP trend clusters were predicted for the entire watershed at the scale of river segments, which are more directly relevant to watershed planning. Among the 975 river segments, 544 (56%) and 431 (44%) were classified as ‘monotonic increase’ and ‘monotonic decrease’, respectively. Furthermore, these predicted TP trend clusters were paired with our previously published total nitrogen (TN) trend clusters, showing that TP and TN both declined in 185 segments (19%) and neither declined in 337 segments (35%). Broadly speaking, large-scale nutrient reduction efforts are underway in many regions to curb eutrophication. Water-quality responses and drivers may differ among systems, but our work provides important new evidence on the effectiveness of management efforts toward controlling point and nonpoint sources.

## 1. Introduction

Many inland and coastal waterbodies are experiencing ecological degradation due to nutrient enrichment [1–3]. This widespread phenomenon has been attributed to excessive nitrogen (N) and phosphorus (P) loads from the watershed, including point and nonpoint sources. As a key component of the land-river-estuary continuum, rivers are often monitored to quantify water-quality loads and trends, which provide important clues for elucidating interacting drivers [4–7].

Explaining riverine water-quality trends has long been an active area of research, but several general challenges need to be highlighted. First, trend studies often focus on a limited number of locations, making conclusions difficult to generalize given the complexity of watersheds. Second, trend studies may not involve all major input sources, leading to potentially inaccurate inferences. Third, regional monitoring networks may not cover the entire watershed and may not represent the spatial scale relevant to management intervention. These challenges are fully elaborated in our prior publication on total N (TN) trends in the Chesapeake Bay watershed (CBW) [8], where we demonstrated that two machine learning approaches, i.e. hierarchical clustering and random forest (RF), can be applied to monitoring data sets to describe the complexity of water-quality changes and elucidate interacting drivers. Specifically, clustering approaches can be used to categorize regional patterns of water-quality trends. RF can further be used to identify potential drivers of water-quality trend clusters and to make predictions for areas where monitoring data do not exist.

In our current work, we applied the clustering and RF approaches to better understand the regional patterns and drivers of total P (TP) load trends in the 166 000 km^2^ watershed of Chesapeake Bay, which is the largest estuary in the United States and one of the most productive in the world. This new work on TP trends is timely for several reasons. First, reductions of TN and TP loads are both crucial for controlling eutrophication. It is well known that nutrient limitation in the Bay exhibits strong spatial and seasonal variations, with P-limitation common along a large proportion of the mainstem in winter-spring months [9, 10]. Therefore, reduction of N alone is insufficient to control algal growth, and dual reductions of N and P are mandated in the Chesapeake Bay total maximum daily load (TMDL) [11]. Second, P differs from N considerably in terms of sources, transport pathways, and transformation mechanisms, making it necessary to conduct a separate analysis on P load patterns and trends. In general, N is dominated by dissolved forms transported in subsurface flows, whereas P is dominated by particulate P attached to sediment particles and transported in surface flows [12, 13]. While N can be permanently lost from the watershed via denitrification, P may accumulate in soils or be stored in rivers and reservoirs via sedimentation [14, 15]. Third, although decades of efforts have been made to manage point sources [16–18] and nonpoint sources [19, 20], P loads have shown mixed, short-term trends across the CBW, with decreasing trends observed at less than half of monitoring stations [21]. This is surprising considering declines in agricultural P surplus are ubiquitous across the CBW and highlights that an internal source of P is being mobilized within the watershed [22], as has been observed in the Mississippi River basin [23]. In regard to the variable trends and paradoxical deviation from the expected input–output relationship, advanced data analysis approaches should be implemented to elucidate interacting drivers, especially considering that much debate surrounds the drivers of P loads in the CBW and the effectiveness of point and nonpoint source nutrient controls.

Consistent with our prior work on TN trends [8], this work on TP trends involved three sequential steps: (1) hierarchical clustering was used to categorize TP trends from 2013 to 2020 at the monitoring stations. This period was chosen to incorporate TP data from recently established stations. (2) RF models were developed to identify likely drivers of the TP trend clusters. (3) TP trend clusters were predicted for the entire CBW (including unmonitored areas) at the scale of river segments (*n* = 975) (figure S1) to infer where TP loads are likely increasing or decreasing. These segments are designated based on river reaches and are more directly relevant to watershed planning.

We emphasize that our current work is not intended for introducing clustering and classification approaches toward understanding regional water-quality patterns and trends, which has already been demonstrated in our prior work on TN trends [8]. Instead, new efforts and contributions of this work include: (1) compilation of watershed and riverine data sets necessary for applying the two machine learning approaches to analyze TP trends, (2) categorization of TP trend clusters across the CBW, (3) development and selection of optimal RF models for capturing the likely regional drivers of TP trend clusters, (4) prediction of TP trend clusters for the entire CBW, including unmonitored areas, and (5) synthesis of TN and TP trend clusters for the entire CBW. For the last aspect, we compared the predicted TP trend clusters from this work and the predicted TN trend clusters from our prior work [8] to identify locations where both nutrients are likely increasing or decreasing. These results provide useful information for prioritization of watershed management toward the dual reduction of N and P under the Bay TMDL.

## 2. Data and methods

### 2.1. Hierarchical clustering

Riverine water quantity and quality has been measured by the non-tidal network (NTN). Flow-normalized loads (FNLs) of TP were reported for 90 NTN stations by Mason *et al* [21] using the Weighted Regressions on Time, Discharge, and Season (WRTDS) method [24]. Depending on the station, the estimation period begins as early as 1985 and ends in 2020. FNLs can better reveal trends by effectively removing the interannual variability in streamflow and have been used for estimating water-quality trends in the CBW [21, 24, 25] and many other regions [5, 6].

We chose to analyze annual TP FNLs in the period of 2013–2020 because 37 stations were added to NTN in 2011–2013. Annual FNLs at each station in the period of 2013–2020 were standardized (mean = 0, s.d. = 1). Hierarchical clustering categorized the standardized FNLs at the 90 stations into two distinct clusters (table S1). The optimal number of clusters (two) was determined by the silhouette method. The results were validated using a re-sampling and re-classifying procedure. More details of hierarchical clustering, including data from three example stations, are described in supporting information (SI, part A).

### 2.2. RF classification

RF was used to infer likely drivers of TP trend clusters—see justification in supporting information (SI, part B1). 13 explanatory variables (called ‘features’) were pre-selected (table 1), because of their documented effects on P loads and trends [26–33]. More details are described in supporting information (SI, part B2). Some features had missing values, thereby reducing the record for RF analysis to 67 NTN stations (table S2).

Like our prior work on TN trends [8], an exhaustive search algorithm was implemented to search for the optimal RF models. To constrain model complexity, the RF models were set to have six or fewer features. Three optimal models were identified based on the out-of-bag (OOB) accuracies. Partial dependence plots were developed to show the marginal effect of each feature. Unlike ANOVA, partial dependence plots from the RF models can provide a simultaneous and more rigorous assessment of the marginal effects of each feature. More details of the exhaustive search algorithm are described in supporting information (SI, part B3).

Consistent with our prior work on TN trends [8], an ensemble model approach was implemented to combine the strengths of the three optimal models. The predicted probability (Pr) associated with the prediction by each model was compared, and the prediction with the highest Pr was selected. The predicted Pr was used as a measure of likelihood, which is classified as ‘high’ (Pr ⩾ 0.667), ‘medium’ (0.5 ⩽ Pr *<* 0.667), or ‘low’ (Pr *<* 0.5) (table S2; figure S9(a)). The selected threshold for the high likelihood (i.e. 0.667) is consistent with the established threshold used for determining whether a trend exists in the WRTDS FNLs [21].

### 2.3. RF prediction

The optimal RF models were used to predict TP trend clusters for the entire CBW, including unmonitored areas. Here we used the river segments (*n* = 975) of the Chesapeake Bay Program Watershed Model, which have a mean area of 168 km^2^ [34] (figure S1). Unlike NTN stations, the river segments do not overlap with each other. In addition, these segments are designated based on river reaches and are more directly relevant to watershed planning.

For each river segment, model features were prepared similarly to the NTN stations, which are shown in figures S10 and S11. Then, the three optimal RF models were applied to predict TP trend clusters using the ensemble approach described above (table S3; figure S9(b)).

Lastly, the predicted TP trend clusters and the published TN trend clusters [8] were synthesized to identify watershed locations where both nutrients are likely increasing or decreasing.

## 3. Results and discussion

### 3.1. Regional patterns of TP trend clusters

**Figure 1.**
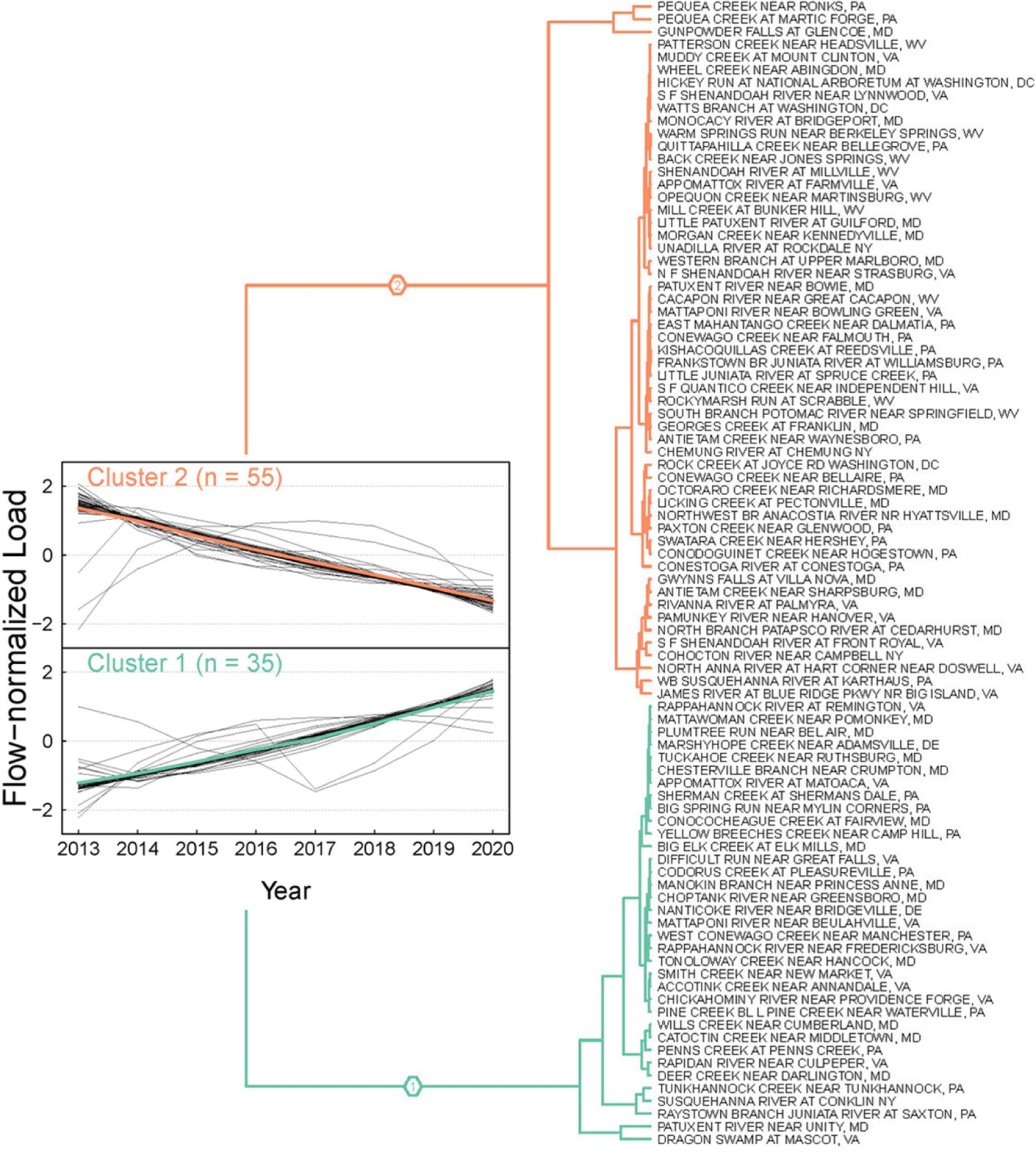
Dendrogram showing the cluster assignment of total phosphorus load trends (2013–2020) at the 90 non-tidal network stations. Trajectories of the two clusters are shown. For each cluster, the cluster-level and station-level trends are shown with colored and gray line, respectively.

Hierarchical clustering categorized the annual FNLs of TP at the 90 monitoring stations into two distinct clusters (figure 1; table S1). These clusters show contrasting trends of TP loads, with cluster 1 (*n* = 35) showing a monotonic increase (i.e. a degrading trend) and cluster 2 (*n* = 55) showing a monotonic decline (i.e. an improving trend). These results were validated by the sensitivity analysis (figure S3).

**Figure 2.**
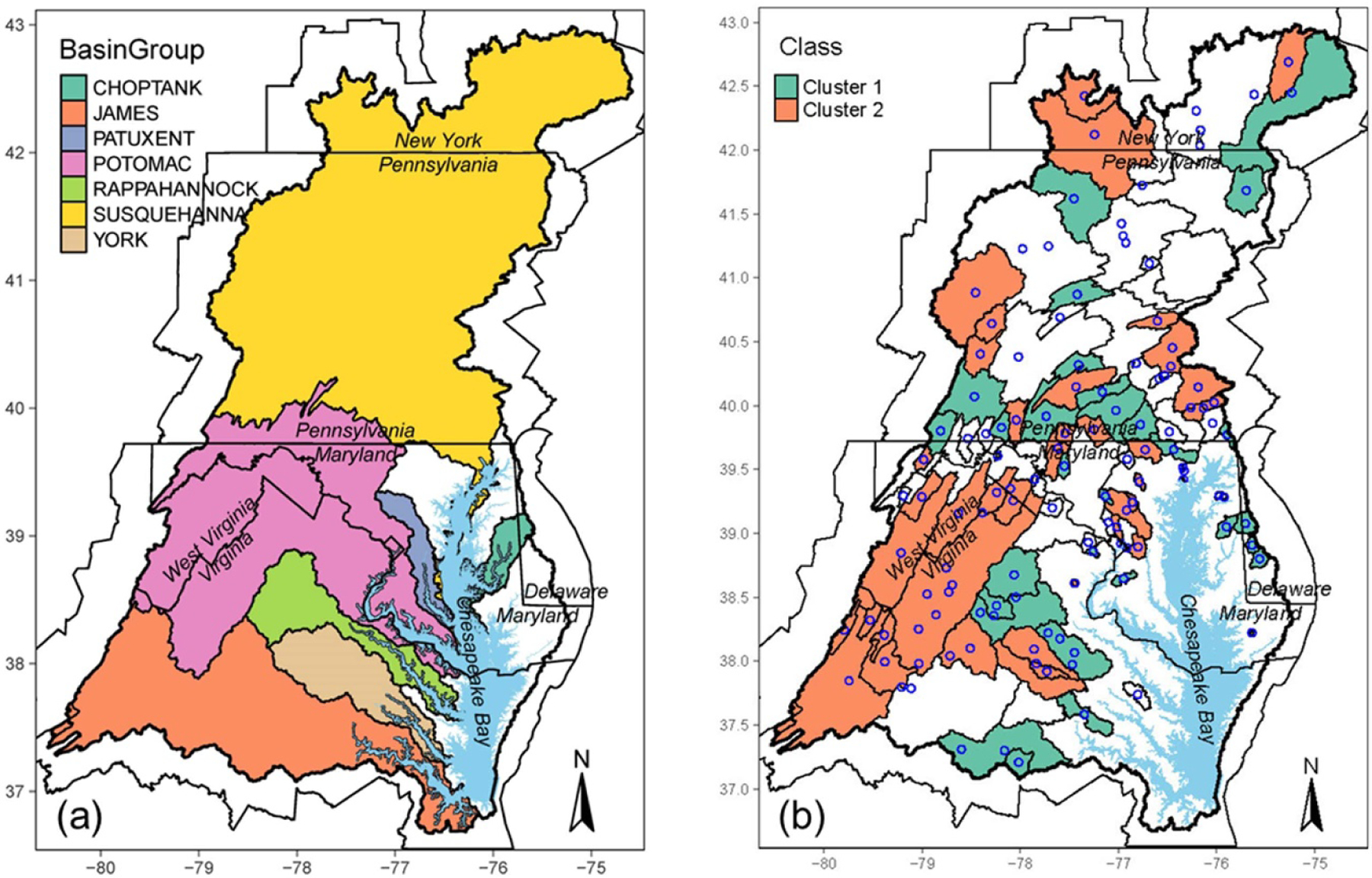
Maps of (a) the Chesapeake Bay major basins and (b) the clusters of total phosphorus trends at the non-tidal network stations. Each open circle in (b) represents one NTN watershed (figure 1).

Spatially, cluster 2 watersheds tend to be major agricultural regions, including the lower Susquehanna River and the Shenandoah Valley (figure 2). It should be noted that many of these areas have had long-term declines in annual TP export, coinciding with long-term decreases in agricultural P surplus and increases in P use efficiency [22]. Cluster 1 watersheds appear to be more scattered but include the Coastal Plain region.

### 3.2. Regional drivers of TP trend clusters

Three optimal RF models were identified (table 2, figure S8). These models have the same overall OOB accuracies and have similar model forms, all including PointSource_MK, Fertilizer_MK, Crop_pct, Natural_pct, and two variables of physiography or geology. Two of the three models have the highest OOB accuracy for cluster 1 (75.0%), while the third model has the highest OOB accuracy for cluster 2 (89.7%). In addition, the ensemble model approach showed better performance than each individual model (figure S9(a)) and its clusters agree well with the direct result from the hierarchical clustering (table S2).

**Figure 3.**
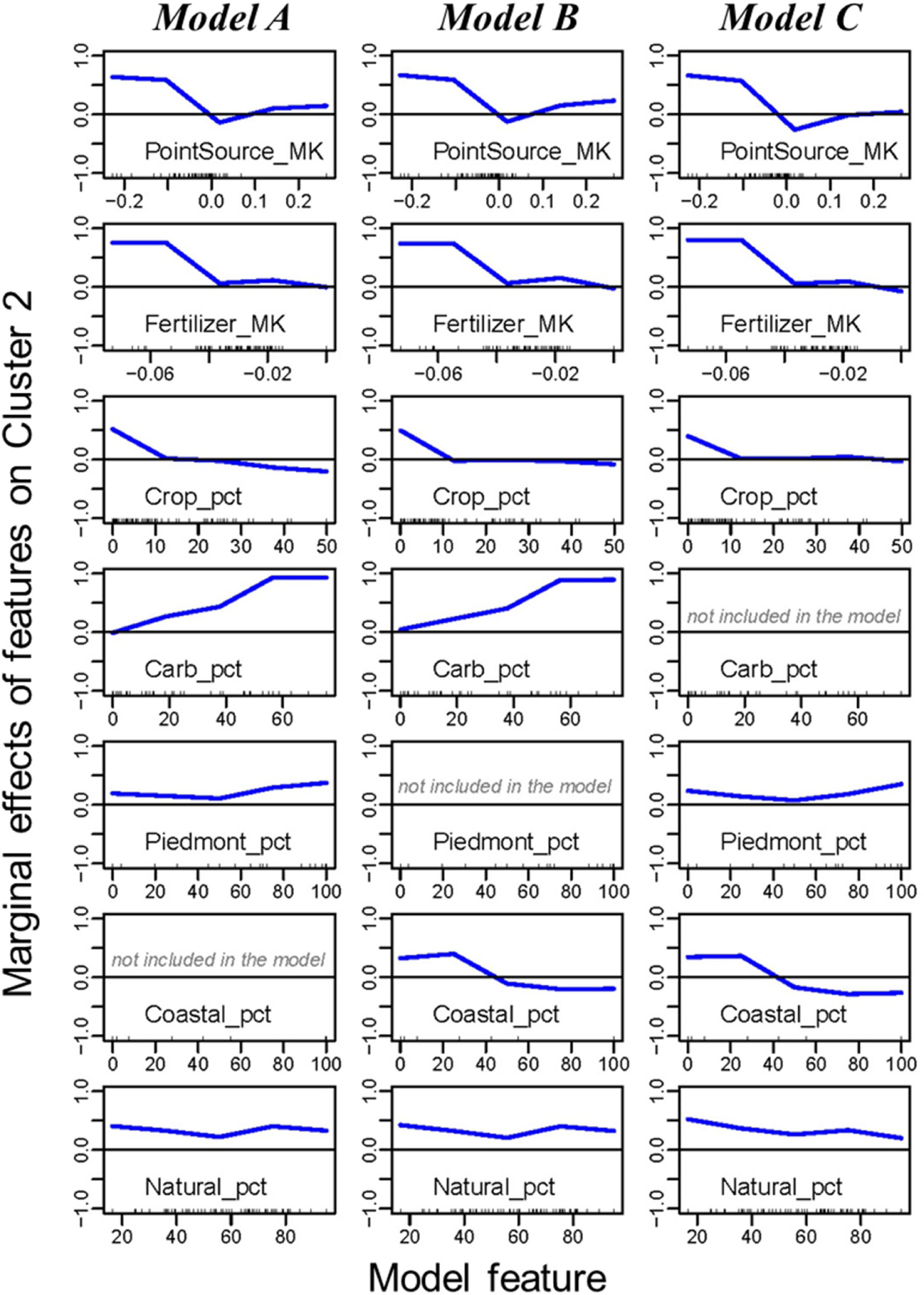
Partial dependence plots for features selected by the three optimal random forest models, showing their marginal effect on the probability of cluster 2.

Partial dependence plots showed the dependence between the target response (i.e. cluster assignment) and each selected feature (figure 3). In terms of sources, cluster 2 has a larger chance to occur in watersheds with large declines in PointSource_MK and Fertilizer_MK. In terms of watershed characteristics, cluster 2 has a larger chance to occur in watersheds with a small Crop_pct, a large Carb_pct, a small Coastal_pct, or a large Piedmont_pct. These relationships have important management implications, which are elaborated in the two sections below. In addition, they are consistent with the base model (using all features) in terms of feature importance (figure S5(a)) and marginal effects (figure S6). The identified features are generally consistent with those determined by ANOVA (table 1; figure S4).

### 3.3. Effects of point and nonpoint source controls

One key message from these results is that both wastewater control and agricultural nutrient management have contributed to water-quality improvement. Declining TP trends (cluster 2) have a larger chance to occur in watersheds with large declines in point source loads (PointSource_MK) and agricultural fertilizer inputs (Fertilizer_MK) (figure 3). Point source reductions were reported to have contributed to water-quality improvement, which can be attributed to implementations of enhanced wastewater treatment technology [22, 26, 35–37]. By contrast, nonpoint sources were reported to have made limited progress [26, 27, 36, 37]. Focusing on recent trends of TP, this work provides encouraging evidence that efforts to reduce point sources (figure S10(a)) and agricultural nonpoint sources (i.e. fertilizer; figure S10(b)) both contributed to water-quality improvement. The inference on declining agricultural nonpoint sources with declining riverine P export resonates with our prior inference with N [8].

We additionally speculate that long-term declines in agricultural nonpoint sources have contributed to mining legacy P from soils, i.e. crop removal exceeding nutrient inputs [22], which are likely quite large due to decades of less efficient use of manure and fertilizer P inputs in agricultural productions [38]. At the same time, we note that agricultural manure inputs have not consistently declined across the CBW due mainly to increased production of poultry (figure S10(c)) and often crop removal has not kept pace in those areas. The increase in manure inputs without corresponding increases in crop yields has caused some of the promising declines in agricultural surplus to begin to reverse [22]. Therefore, management attention on agricultural areas will be crucial to achieve P reduction goals, especially considering that declining TP trends are more difficult to occur in areas with a large Crop_pct (figure 3). This may be attributed to higher inputs of P in agricultural areas [26, 33, 35] and long-term accumulations of P applications that exceeded crop needs [30, 38–40]. In the face of climate change, continued and even stronger reductions of agricultural nonpoint sources will be critical because projected increases in rainfall and runoff will likely exacerbate the loss of particulate P to surface water [41].

### 3.4. Effects of watershed physical properties

Watershed physical properties are also important factors affecting the TP trend clusters. Although watershed settings cannot be directly managed, these identified effects provide important clues on P transport and storage and can help facilitate spatially targeted management.

Declining TP trends (cluster 2) have a larger chance to occur in watersheds with a large Carb_pct (figure 3). One likely explanation is the reduction of P availability and export in the carbonate terrain due to the precipitation of phosphate [42] and less accumulation of legacy P in agricultural fields [38]. In our prior work on TN trends [8], a similar effect of Carb_pct was noted, which was attributed to the relatively quick infiltration and groundwater transport of N in this terrain [26].

In contrast, declining TP trends (cluster 2) have a smaller chance to occur in watersheds with a large Coastal_pct (figure 3). Coastal Plain areas, particularly the Delmarva Peninsula, have high P losses in runoff from P-rich, agricultural soils, which are a result of historical application of poultry manure at rates greatly exceeding the amounts necessary for crop growth [22, 30, 39, 40]. Consequently, these areas are expected to take a longer time to achieve improvements [43–45], unless significant efforts are made to remove legacy P. Indeed, many major crop producing regions of the country have near zero or negative annual P cropland balances without compromising crop yields [38]. In our prior work on TN trends [8], a similar effect of Coastal_pct was noted, which was attributed to legacy N in groundwater [46, 47]. In contrast, P tends to bind to soil particles under natural conditions. Thus, soil conservation measures (e.g. cover crops, riparian buffers, and conservation tillage) may help limit the delivery of legacy P from the landscape to streams [30]. However, such practices can often exacerbate P losses by promoting P accumulation and stratification in upper soil layers and hence more mobilization of P during storm and snowmelt events [23], including in the CBW and elsewhere [35, 48].

### 3.5. Predicted TP trend clusters for the CBW

**Figure 4.**
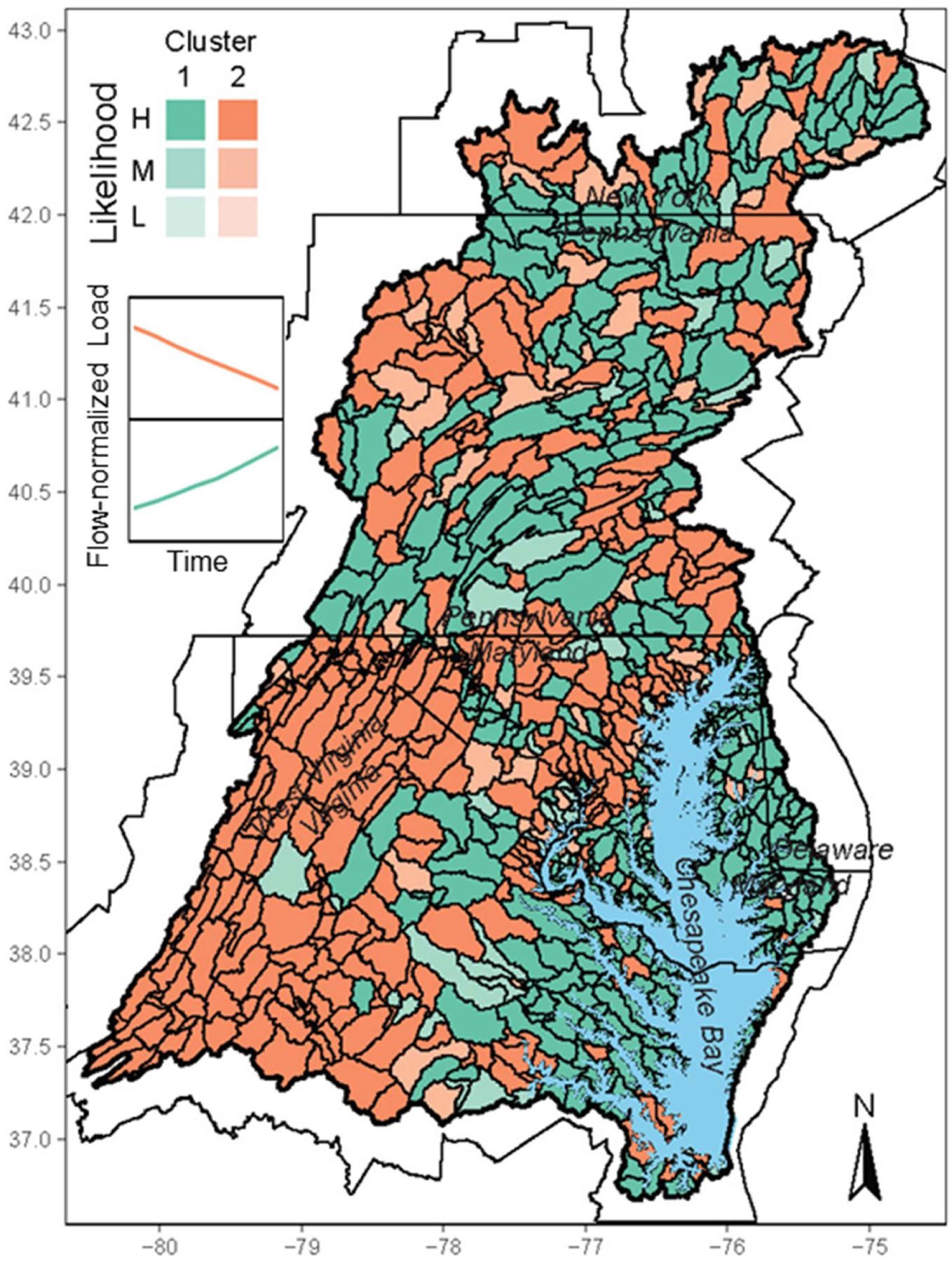
Map of total phosphorus trend clusters for the Chesapeake Bay watershed at the scale of river segments, as predicted by the random forest ensemble model. For each cluster, dark color indicates high likelihood (≥ 0.667), whereas light color indicates low likelihood (< 0.5). Inset shows the temporal trend of each cluster.

The optimal RF models were used to predict TP trend clusters for the entire CBW. Among the 975 river segments, 544 (56%) and 431 (44%) were predicted to be cluster 1 and cluster 2, respectively (figure 4; table S3). Among these predictions, 85% had high likelihood and 15% had medium likelihood. Spatially, cluster 2 segments are generally concentrated in the lower Susquehanna River (east of the mainstem), the Shenandoah Valley/Great Appalachian Valley, and the upper Western Shore. Cluster 1 segments are in the northern part of the CBW, western Pennsylvania, and Coastal Plain areas on both sides of the Bay. Lastly, the two clusters differ in terms of proximity to the Bay, with cluster 2 segments dominating in areas further upstream in the watershed and cluster 1 segments dominating in areas close to the Bay (figure S12). This pattern of P clusters contrasts with our published N clusters, which showed cluster 2 dominance in areas adjacent to the Bay [8].

These predictions are concerning from a management perspective. A majority of the segments (56%) have increasing TP trends (cluster 1) and most of them (78%) have high likelihoods. In addition, cluster 1 segments are generally closer to the Bay than cluster 2 segments, which may hinder the restoration progress because changes in areas closer to and directly connected to the tidal waters should have a stronger impact on the estuary than upland areas due to limited opportunities for retention and mitigation [37, 49, 50]. Optimizing the management and use of P in farm operations in the Coastal Plain, as well as controlling erosion, will be key for reversing the upward trends of TP.

It should be noted that a small fraction of the river segments (i.e. 18%) are beyond the range of the NTN watersheds in terms of watershed area, and their predictions may be more uncertain. Indeed, for those segments in the extrapolated range, 77% have high likelihoods and 23% have medium likelihoods. In comparison, for segments within the range of the NTN watersheds, 86% have high likelihoods and only 14% have medium likelihoods. Nonetheless, the predictions for the river segments are not expected to be significantly impacted by watershed size for several reasons: (1) most river segments (i.e. 82%) are within the range of the NTN watersheds, which were used in the RF model development, (2) we have purposefully chosen to use area-normalized values for the nutrient input variables, which are not dependent on watershed size, (3) our response variable (i.e. trend cluster) is not dependent on watershed size, which differs from trend magnitude, and (4) Area_km2 was not selected as a significant variable by RF, implying that watershed size was not a key driver for the trend clusters of the NTN watersheds and therefore, watershed size is unlikely to be a major source of bias for the river-segment predictions.

### 3.6. Synthesis of predicted TP and TN trend clusters

**Figure 5.**
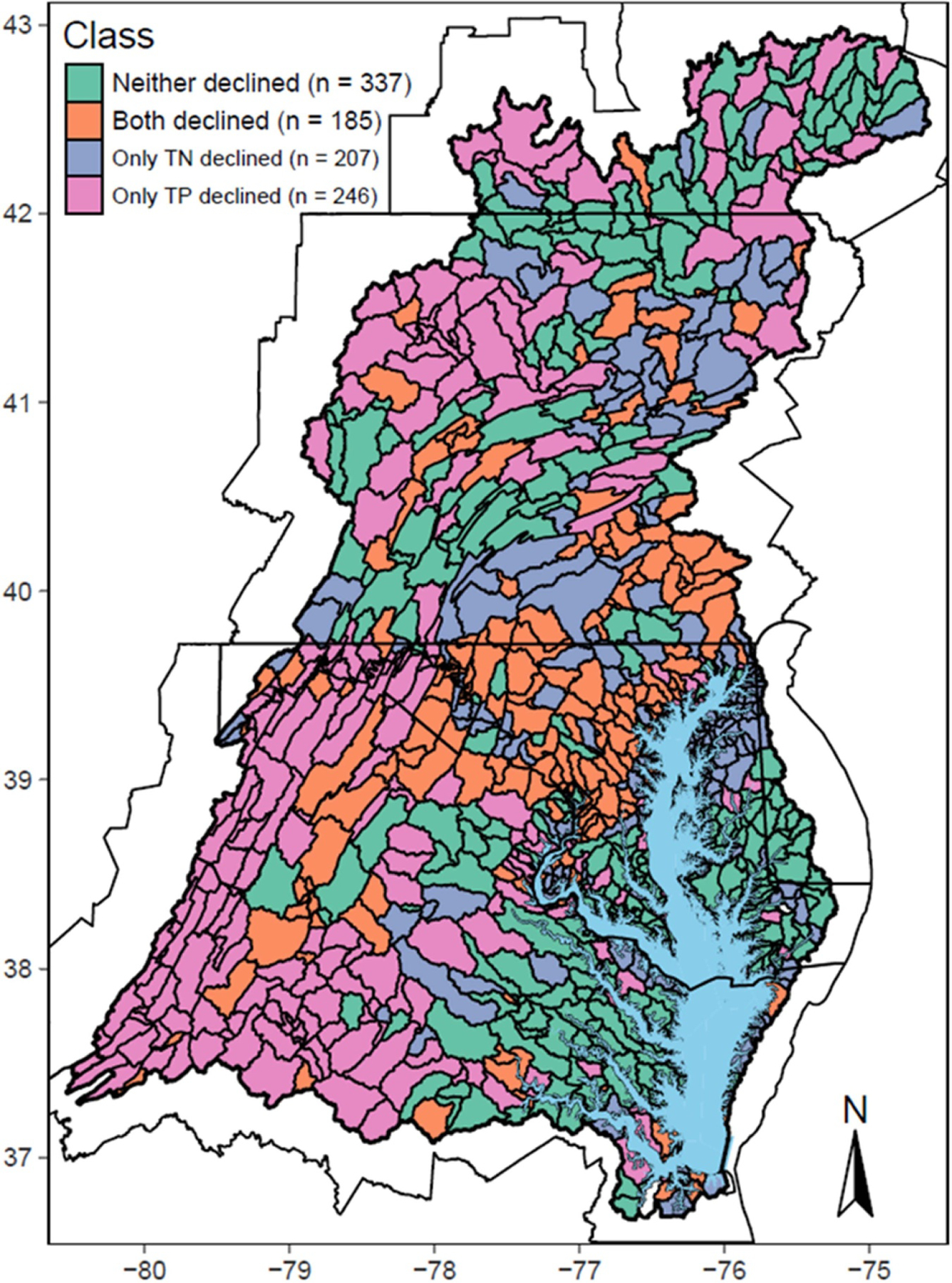
Map of total phosphorus and total nitrogen trend clusters for the Chesapeake Bay watershed at the scale of river segments. For simplicity, likelihood estimates are not shown.

Dual reductions of N and P are mandated in the Bay TMDL. Therefore, we further synthesized the predicted TP trend clusters with previously published TN trend clusters [8] to provide information toward guiding targeted watershed management (figure 5). Among the 975 river segments, TP and TN both declined in 185 segments (19% of the total). They are concentrated in the lower Susquehanna watershed, the upper Western Shore, and the Shenandoah Valley. By contrast, neither TP nor TN declined in 337 segments (35%), which is almost twice that of the ‘both declined’ group. These segments are in the northern part of the watershed, western Pennsylvania, and the low-lying Coastal Plain areas. The remaining watershed is comprised of 453 segments (46%), with 207 segments having only TN declines and 246 segments having only TP declines, which may potentially lead to enhanced N limitation and enhanced P limitation, respectively. Altogether, about two thirds of the river segments in the watershed had declines in TN, TP, or both. Despite such progress, continued nutrient reductions, especially from agricultural nonpoint sources, are imperative in order to achieve the TMDL targets and improve water quality in the estuary.

## 4. Conclusions

To better understand the regional patterns and drivers of TP trends across the CBW, we compiled and analyzed TP loads data at 90 NTN stations using a combination of clustering and RF approaches. These stations were categorized into two distinct clusters of short-term (2013–2020) TP load trends, i.e. monotonic increase (*n* = 35) and monotonic decline (*n* = 55). RF models provided evidence that reductions in point sources and agricultural nonpoint sources (i.e. fertilizer) both contributed to water-quality improvement, thereby demonstrating the effectiveness of nutrient management and the importance of continuing such efforts. In addition, declining TP trends have a larger chance to occur in carbonate areas but a smaller chance in Coastal Plain areas, with the latter likely reflecting the effect of legacy P. To provide spatially explicit information, TP trend clusters were predicted for the entire CBW at the scale of river segments, which are more directly relevant to watershed planning. Among the 975 river segments, 544 (56%) and 431 (44%) were classified as ‘monotonic increase’ and ‘monotonic decrease’, respectively. These predicted TP trend clusters were further compared with our previously published total nitrogen (TN) trend clusters, showing that TP and TN both declined in 185 segments (19%) and neither declined in 337 segments (35%).

We recognize that TP trend estimates will be updated in two years for the NTN stations, and hence we recommend a re-evaluation of the trend patterns and drivers for a longer analysis period (2013–2022 vs. 2013–2020) when new trend estimates are available. In addition, our RF models were constrained to explanatory variables currently available for the NTN stations, which satisfactorily captured the regional patterns of TP with no more than six features. However, the performance may further improve with additional features, which include but are not limited to management practices [20, 51], legacy P [43, 44], landscape configuration [52, 53], and reservoir retention [54–56]. Lastly, we note that the river-segment predictions from the RF models should be interpreted with caution if any prediction does not have a high likelihood or if any river segment has a watersheds area that is beyond the range of the NTN watersheds.

This research demonstrates the importance of sustaining regional water-quality monitoring networks and the usefulness of advanced data analysis approaches toward generating new insights from the monitoring records regarding spatial patterns of water-quality trends (i.e. clustering) and relative impacts of natural and anthropogenic drivers (i.e. classification). Broadly speaking, large-scale nutrient reduction efforts are underway in many regions to curb eutrophication, including Northern Gulf of Mexico, Tampa Bay, and Baltic Sea [1–3]. Water-quality responses and drivers may differ among systems, but our work provides important new evidence on the effectiveness of management efforts toward controlling point and nonpoint sources.

## Supplementary Material

Supplement1

Supplement2

## Figures and Tables

**Table 1.** The model features and their statistical difference between the clusters of total phosphorus load trends. Each feature was separately evaluated against the response variable using the one-way analysis of variance (ANOVA) test. A feature is statistically different among the two clusters if the reported p-value is less than 0.1 (bold).

Feature	Definition	*p*-value
Area_km2	Watershed area, km^2^	0.30
Crop_pct	Cropland, percent (median of 2013–2019 values)	0.13
Natural_pct	Natural land, percent (median of 2013–2019 values)	0.96
Carb_pct	Carbonate geology, percent	**0.0042**
Coastal_pct	Coastal Plain physiography, percent	**0.015**
Piedmont_pct	Piedmont physiography, percent	0.99
ValleyRidge_pct	Valley and Ridge physiography, percent	**0.030**
BlueRidge_pct	Blue Ridge physiography, percent	0.18
Appalachian_pct	Appalachian physiography, percent	0.98
PointSource_MK	Mann–Kendall (MK) trend of point source load between 2010 and 2019	0.16
Fertilizer_MK	MK trend of agricultural fertilizer between 1985 and 2019	**0.025**
Manure_MK	MK trend of manure between 1985 and 2019	0.68
AgSurplus_MK	MK trend of agricultural surplus between 1985 and 2019	0.43

**Table 2.** The three optimal random forest models, as selected by the exhaustive search algorithm. The first two models had the highest out-of-bag (OOB) accuracy for cluster 1, while the third model had the highest OOB accuracy for cluster 2.

		OOB accuracy, percent		
Model	Model form	Overall	Cluster 1	Cluster 2
A	Fertilizer_MK + Crop_pct + PointSource_MK + Natural_pct + Carb_pct + Piedmont_pct	**79.1**	**75.0**	82.1
B	Fertilizer_MK + Natural_pct + PointSource_MK + Crop_pct + Carb_pct + Coastal_pct	**79.1**	**75.0**	82.1
C	Fertilizer_MK + Coastal_pct + Crop_pct + PointSource_MK + Piedmont_pct + Natural_pct	**79.1**	64.3	**89.7**

## Data Availability

Source data are described in the Data and Methods. Data on clustering results, model features, and model predictions are presented in the supplemental tables.
