# Supplementary material for: Effects of point and nonpoint source controls on total phosphorus load trends across the Chesapeake Bay watershed, USA
Source: Environ Res Lett. Author manuscript; Available in PMC 2024 Dec 1. (PMC11457064; doi:10.1088/1748-9326/ad0d3c)
Supplement: Supplement1 [file NIHMS1999647-supplement-Supplement1.pdf]

SUPPORTING INFORMATION

Effects of point and nonpoint source controls on total phosphorus load trends across the Chesapeake Bay watershed, USA

Qian Zhang[1*], Joel T. Bostic[2,3], Robert D. Sabo[4]

[1] University of Maryland Center for Environmental Science, Annapolis, Maryland, USA

[2] University of Maryland Center for Environmental Science, Frostburg, Maryland, USA

[3] Garrett College, McHenry, Maryland, USA

[4] U.S. Environmental Protection Agency, Office of Research and Development, Washington D.C., USA

* Corresponding author:qzhang@chesapeakebay.net

TEXT

Part A. Details on hierarchical clustering

Hierarchical clustering was used to categorize the tandardized FNLs at 90 NTN stations into distinct clusters. Annual FNLs at three example stations are presented in **Figure S2 (a-c)**. Annual FNLs at each station in the period of 2013-2020 were standardized to have a mean of zero and a standard deviation of one to facilitate the comparison of trends as opposed to magnitudes. Among the three example stations, the standardized FNLs show monotonic declines in Patuxent River and Pamunkey River but a monotonic increase in Choptank River (**Figure S2, d-f**).

Dissimilarity between the standardized FNLs at individual stations was measured using the Euclidean distance and the linkage between clusters was determined using Ward's minimum variance method [1]. The Euclidean distance between two time series of FNLs is calculated as the square root of the sum of their squared differences in each year between 2013 and 2020. Among three example stations (**Figure S2, g-i)**, Patuxent River and Pamunkey River are most similar to each other, while Patuxent River and Choptank River are most dissimilar.

The hierarchical clustering was performed in R using the "hclust" function in the "stats" package [2]. The optimal number of clusters (two) was determined by the silhouette method [3] (**Table S1**).

The "resampling and re-classifying procedure" of Wolfe, *et al.* [4] was adopted to validate the clustering results. Specifically, the 90 NTN stations were divided into 10 non-overlapping subsets and 10 iterations of the cluster analysis were run (**Figure S3**). In each iteration, one subset (9 stations) was removed, and the remaining subsets (81 stations) were analyzed using the same procedure. The number of clusters was always

set at two to be consistent with the full-data analysis. The assigned clusters from these iterations were compared with those from the full-data analysis to assess agreement (**Figure S3**).

Part B. Details on the random forest (RF) classification

B1. Advantages of RF

RF was chosen to infer likely drivers of TP trends clusters for four reasons. First, RF has been successful as a classification approach [5], which is appropriate for our work as the trend clusters are a categorical variable. Second, RF are robust to data outliers and can efficiently handle non-linear variables [6, 7]. Third, RF can produce generally more stable results than other tree-based approaches due to its bagging algorithm [6]. Fourth, RF can reveal the impact of each explanatory variable by quantifying its relative importance and marginal effect [6, 7].

B2. Model features

A total of 13 explanatory variables ("features") were considered for predicting the TP trend clusters (**Table 1**). These candidate features fall into the categories of watershed area (n = 1), land use (n = 2), geology (n = 1), physiography (n = 5), and Mann-Kendall trend slope of major P sources (n = 4). For each station, land use variables, expressed in percent, were quantified using the CBP Chesapeake Assessment Scenario Tool (CAST) [8]. Geology variables (in percent) were obtained from the U.S. conterminous wall-to-wall anthropogenic land use trends dataset [9]. Physiography variables (in percent) were obtained from the U.S. Geological Survey [10].

In addition, P source data were obtained from CAST for applicable river segments, which were aggregated to obtain the annual mass rate (in kg P/year) for each major source for each NTN station. These annual time series were scaled by their respective long-term means to facilitate comparison of interannual trends. Mann-Kendall trends [11] were computed for point

source load ("PointSource_MK"), manure (livestock manure + direct deposition of manure onto pasture; "Manure_MK"), agricultural fertilizer ("Fertilizer_MK"), and agricultural surplus ("AgSurplus_MK"; *AgSurplus = manure + agricultural fertilizer – crop removal*). For point source load, MK trends were computed for the 2010-2019 period, similar to the period of the response variable (i.e., trend cluster). For nonpoint sources, MK trends were computed for the 1985-2019 period to allow for a 20-year extension that may account for lag effects [12-15]. Among six options of lags tested (i.e., 25-year, 20-year, 15-year, 10-year, 5-year, and 0-year), the 25-year lag was determined to be most effective in modeling the response variable.

Each candidate feature was separately evaluated against the response variable using the one-way analysis of variance (ANOVA) test to examine if the feature had significant differences among the clusters (**Table 1; Figure S4**). The correlation between the features were assessed using the Spearman's correlation [16]. The features generally had low Spearman's correlation (**Figure S5b**), suggesting that they can provide unique information toward explaining the trend clusters.

All candidate features were used to build a base model using the "train" function in the "caret" R package [17] to quantify the relative importance of the features (**Figure S5a**). For the base RF model (**Figure S6**) and subsequently selected RF models, partial dependence plots were developed to show the marginal effect of each feature, i.e., the relationship between the predicted response and each feature while holding all other features constant.

B3. Exhaustive search algorithm

To determine the optimal RF models, an exhaustive search algorithm was developed and implemented in R to evaluate all possible combinations of the candidate features. To constrain model complexity, this algorithm was set to allow no more than six features. Experimentation

showed that model accuracy reached a plateau at about five features and additional features failed to improve model accuracy (see **Figure S7** for an example).

For each candidate model form, RF was run using the “randomForest” function in the “randomForest” R package [18] with the same parameterization (i.e., ntree = 1,000, nodesize = 1, seed = 123), and the model performance was validated using the out-of-bag (OOB) accuracy (i.e., 100 – OOB error) [6]. Specifically, the original data were resampled to form bootstrap samples and used for model training. Records that were not contained in the bootstrap samples (called “OOB samples”) were used for model validation by calculating the OOB error (i.e., the proportion of incorrect classifications) [6].

Three optimal models with the highest OOB accuracies were identified (**Table 2**), all of which showed better performance than the base RF model (**Figure S8a**). Furthermore, leave-one-out cross-validation (LOOCV) was conducted, which also shows better performance by the optimal models than the base model (**Figure S8b**).

References

1. Ward, J. H., Hierarchical grouping to optimize an objective function. *Journal of the American Statistical Association* **1963,** *58*, (301), 236-244.
2. R Core Team *R: A language and environment for statistical computing*, R Foundation for Statistical Computing, Vienna, Austria. http://www.R-project.org/: 2019.
3. Kaufman, L.; Rousseeuw, P. J., *Finding groups in data: An introduction to cluster analysis*. John Wiley & Sons, Inc: 1990.
4. Wolfe, J. D.; Shook, K. R.; Spence, C.; Whitfield, C. J., A watershed classification approach that looks beyond hydrology: application to a semi-arid, agricultural region in Canada. *Hydrol. Earth Syst. Sci.* **2019,** *23*, (9), 3945-3967.
5. Biau, G.; Scornet, E., A random forest guided tour. *Test* **2016,** *25*, (2), 197-227.
6. Hastie, T.; Tibshirani, R.; Friedman, J., *The elements of statistical learning: Data mining, inference, and prediction*. Second Edition ed.; Springer-Verlag New York: 2009.
7. Breiman, L., Random Forests. *Machine Learning* **2001,** *45*, 5-32.
8. Chesapeake Bay Program Chesapeake assessment and scenario tool (CAST) version 2017d. https://cast.chesapeakebay.net/Documentation/ModelDocumentation.
9. Falcone, J. A. *U.S. conterminous wall-to-wall anthropogenic land use trends (NWALT), 1974–2012*; Data Series 948; U.S. Geological Survey: Reston, VA, 2015.
10. Wieczorek, M. E.; Jackson, S. E.; Schwarz, G. E., Select attributes for NHDPlus version 2.1 reach catchments and modified network routed upstream watersheds for the conterminous United States (ver. 3.0, January 2021). In Survey, U. S. G., Ed. U.S. Geological Survey data release: U.S. Geological Survey, 2018.

11. Kendall, M. G., *Rank correlation methods*. 4th Edition ed.; Oxford University Press: London, UK, 1975.

12. Van Meter, K. J.; McLeod, M. M.; Liu, J.; Tenkouano, G. T.; Hall, R. I.; Van Cappellen, P.; Basu, N. B., Beyond the mass balance: Watershed phosphorus legacies and the evolution of the current water quality policy challenge. *Water Resour. Res.* **2021,** *57*, (10), e2020WR029316.

13. Jarvie, H. P.; Sharpley, A. N.; Spears, B.; Buda, A. R.; May, L.; Kleinman, P. J., Water quality remediation faces unprecedented challenges from "legacy phosphorus". *Environ. Sci. Technol.* **2013,** *47*, (16), 8997-8.

14. Chen, D.; Hu, M.; Guo, Y.; Dahlgren, R. A., Influence of legacy phosphorus, land use, and climate change on anthropogenic phosphorus inputs and riverine export dynamics. *Biogeochemistry* **2014,** *123*, (1-2), 99-116.

15. Stackpoole, S. M.; Stets, E. G.; Sprague, L. A., Variable impacts of contemporary versus legacy agricultural phosphorus on US river water quality. *Proc. Natl. Acad. Sci. U. S. A.* **2019,** *116*, (41), 20562-20567.

16. Spearman, C., The proof and measurement of association between two things. *Am. J. Psychol.* **1904,** *15*, 72-101.

17. Kuhn, M.; Wing, J.; Weston, S.; Williams, A.; Keefer, C.; Engelhardt, A.; Cooper, T.; Mayer, Z.; Kenkel, B.; R Core Team; Benesty, M.; Lescarbeau, R.; Ziem, A.; Scrucca, L.; Tang, Y.; Candan, C.; Hunt, T. *caret: Classification and Regression Training*, 2020.

18. Liaw, A.; Wiener, M., Classification and regression by randomForest. *R News* **2002,** *2*, (3), 18-22.

TABLES

Table S1. The Chesapeake Bay Non-Tidal Network (NTN) stations and their total phosphorus trend clusters determined from the hierarchical cluster analysis.

Table S2. The Chesapeake Bay Non-Tidal Network (NTN) stations as well as their total phosphorus trend clusters, explanatory variables (features), and RF model predictions of the trend clusters.

Table S3. The Chesapeake Bay Watershed Model's river segments as well as their explanatory variables (features) and RF model predictions of the total phosphorus trend clusters.

Note: Tables S1-S3 are provided in a separate spreadsheet file.

FIGURES

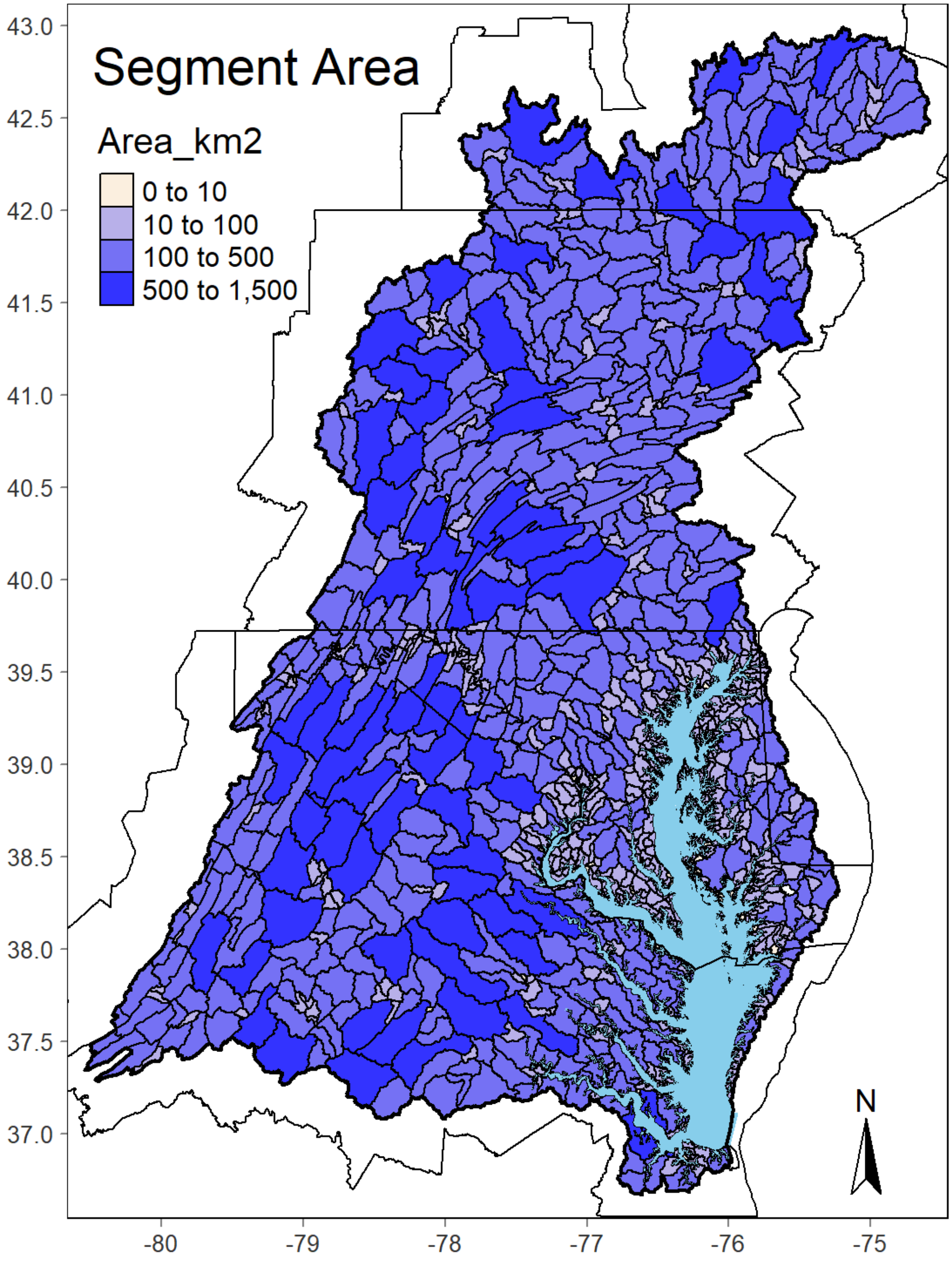

Figure S1. Map of the Chesapeake Bay watershed, showing the river segments (n = 979) of the Chesapeake Bay Watershed Model [8].

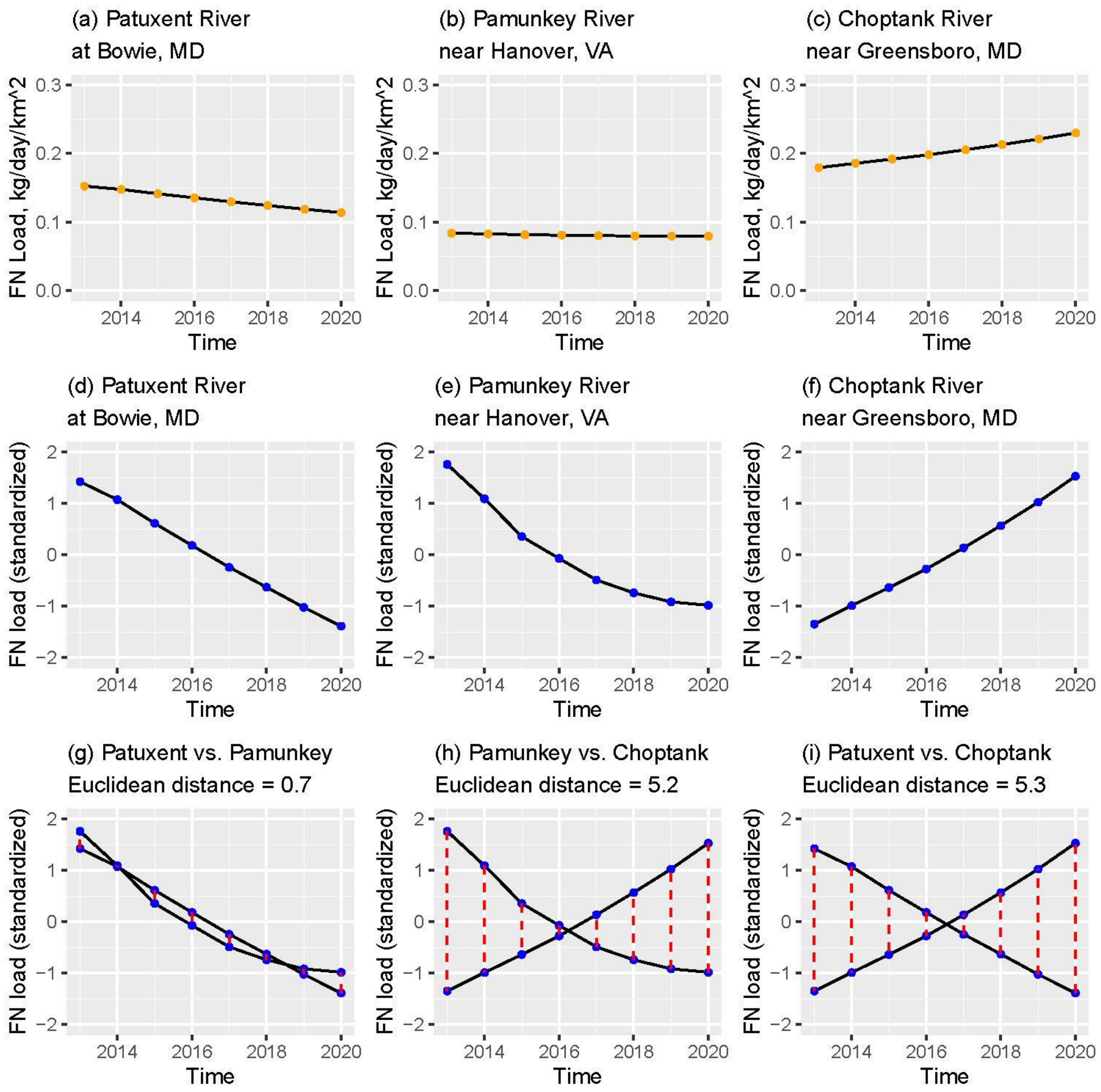

Figure S2. Time series of flow-normalized (FN) total phosphorus loads at three selected monitoring stations, showing (a-c) original loads, (b-f) standardized loads, and (g-i) Euclidean distances of the standardized loads for each pair of stations. The Euclidean distance between two time series of standardized load was calculated as the square root of the sum of the squared differences (dashed, red lines) for each year. Based on the Euclidean distances, Patuxent River and Pamunkey River are most similar to each other, whereas Patuxent River and Choptank River are most dissimilar.

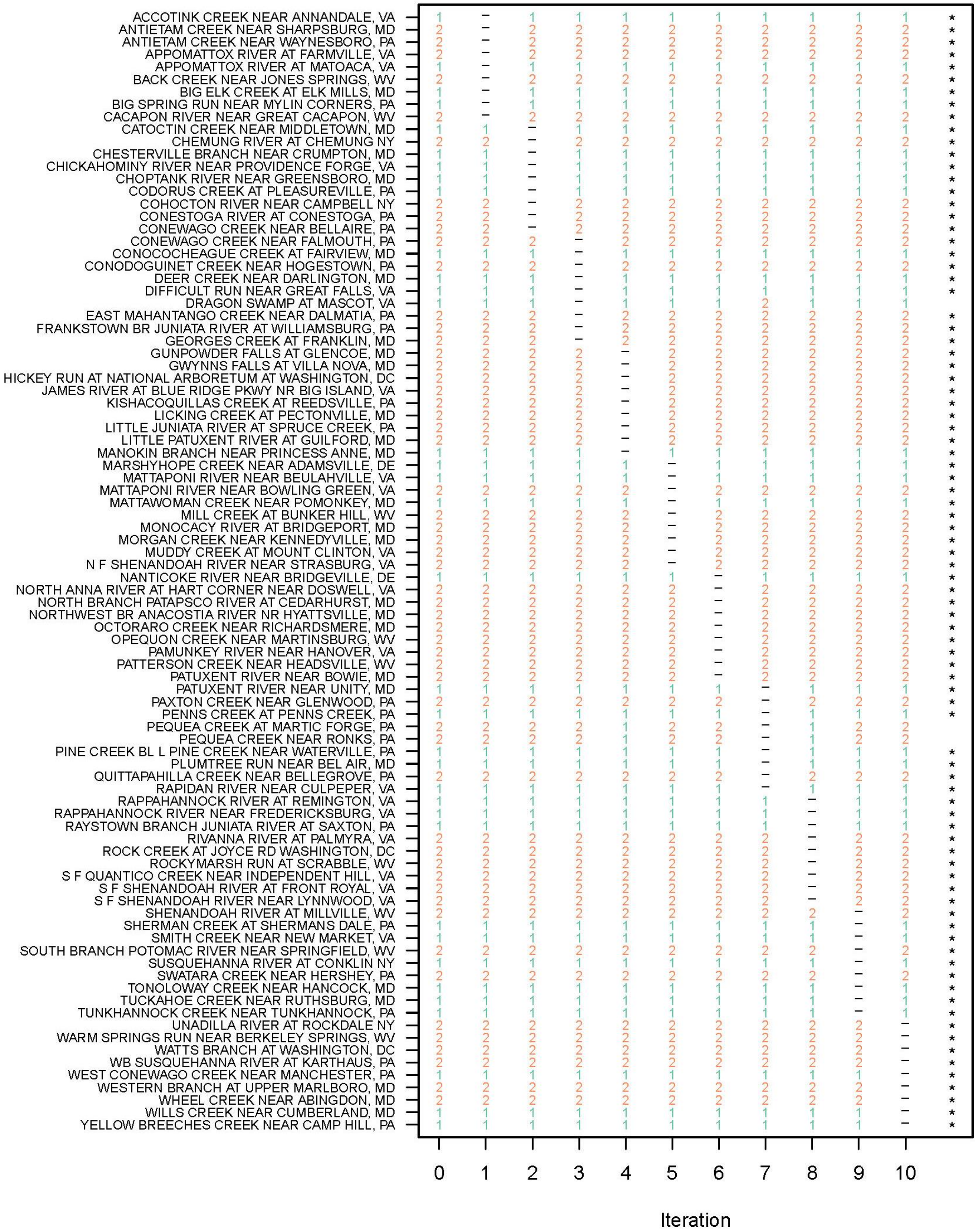

Figure S3. Sensitivity of the hierarchical cluster analysis. "0" represents the full-data cluster analysis, whereas "1" to "10" represent iterations where 1/10 of stations (9 stations) were iteratively excluded. "*" indicates identical cluster assignments among all iterations.

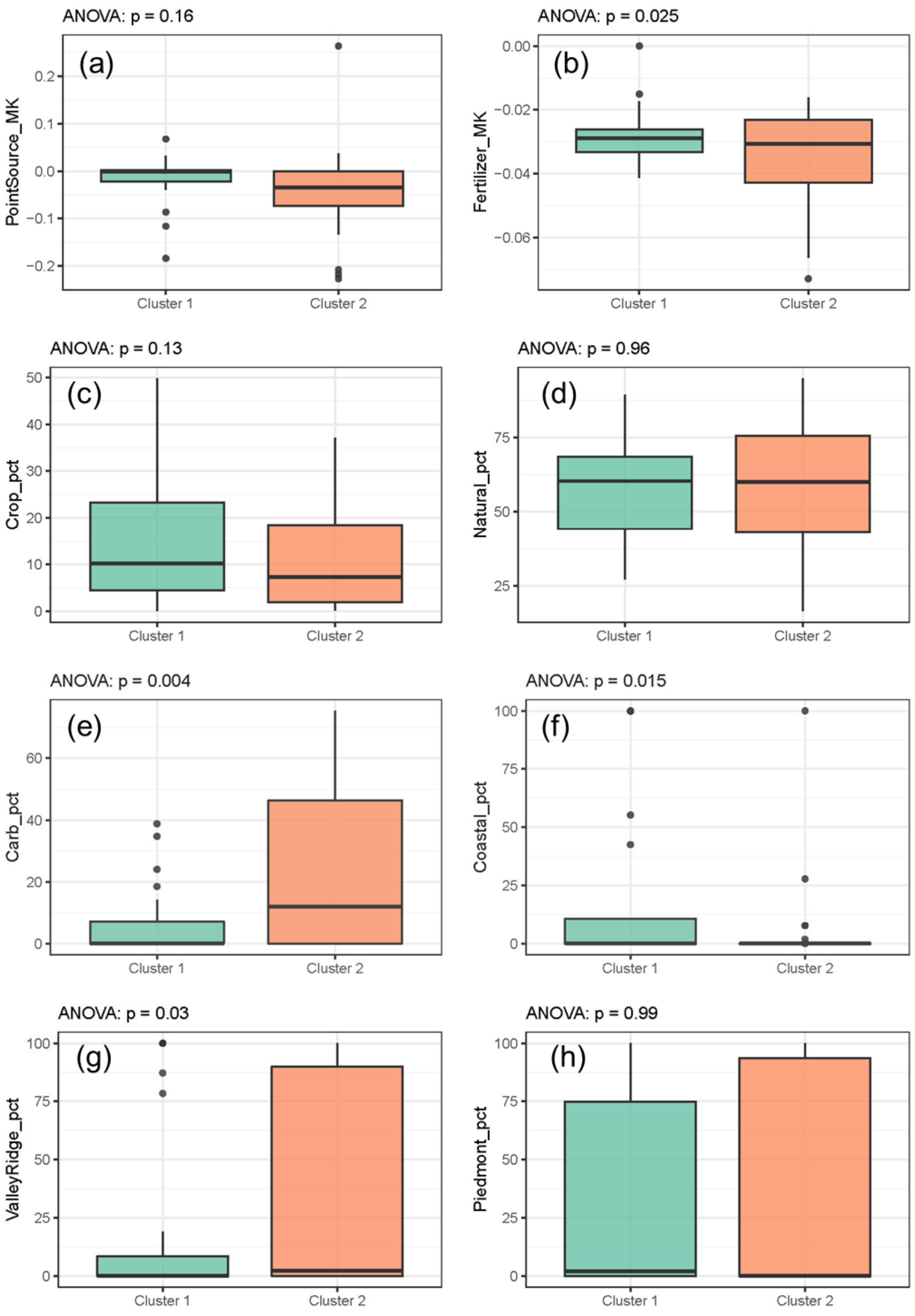

Figure S4. Boxplots comparing the distribution of selected features between the two clusters. The significance of each feature from the ANOVA analysis is provided. A feature (y-axis label) is statistically different between the two clusters if the reported p-value is less than 0.1.

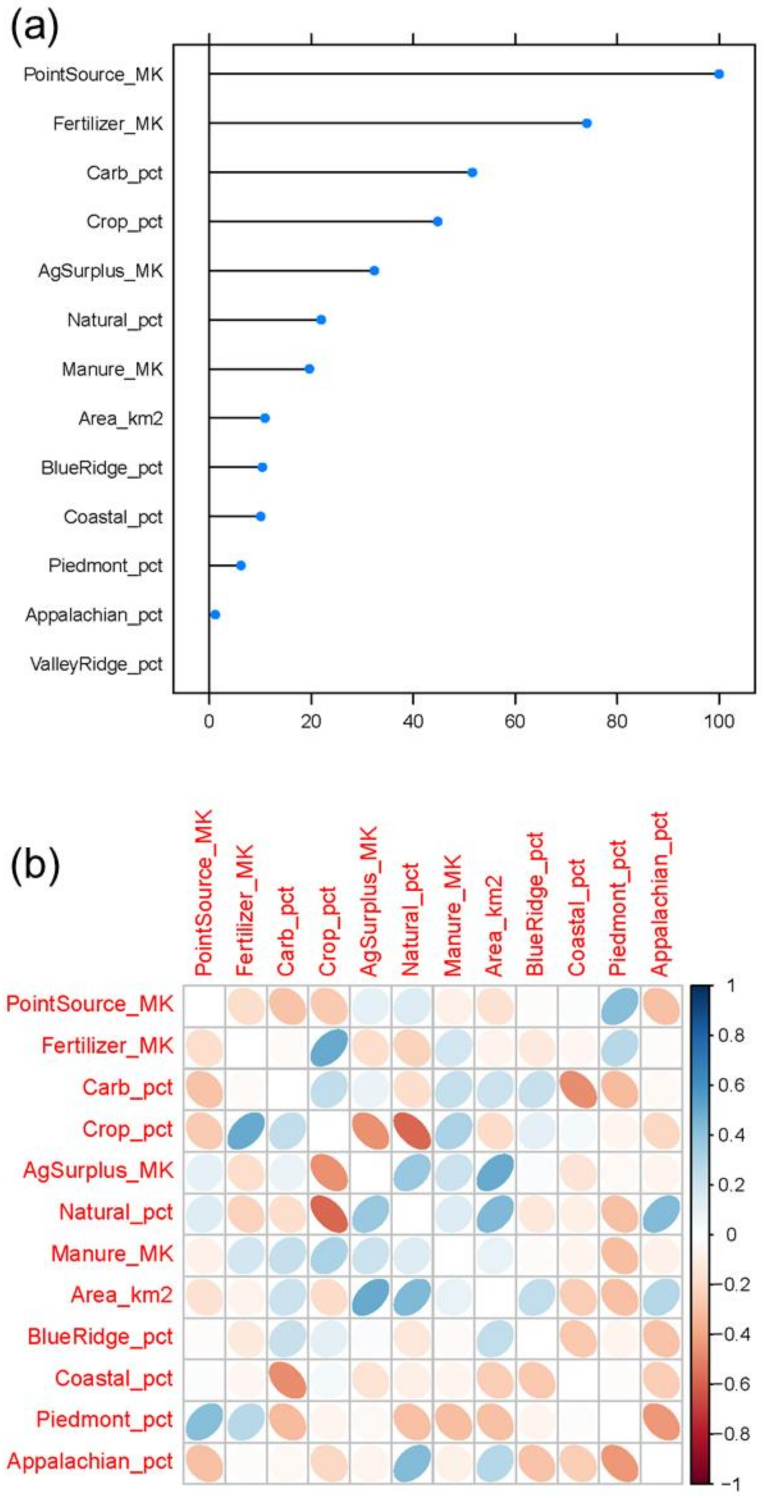

Figure S5. Candidate features in the base random forest model: (a) variable important plot and (b) correlation plots.

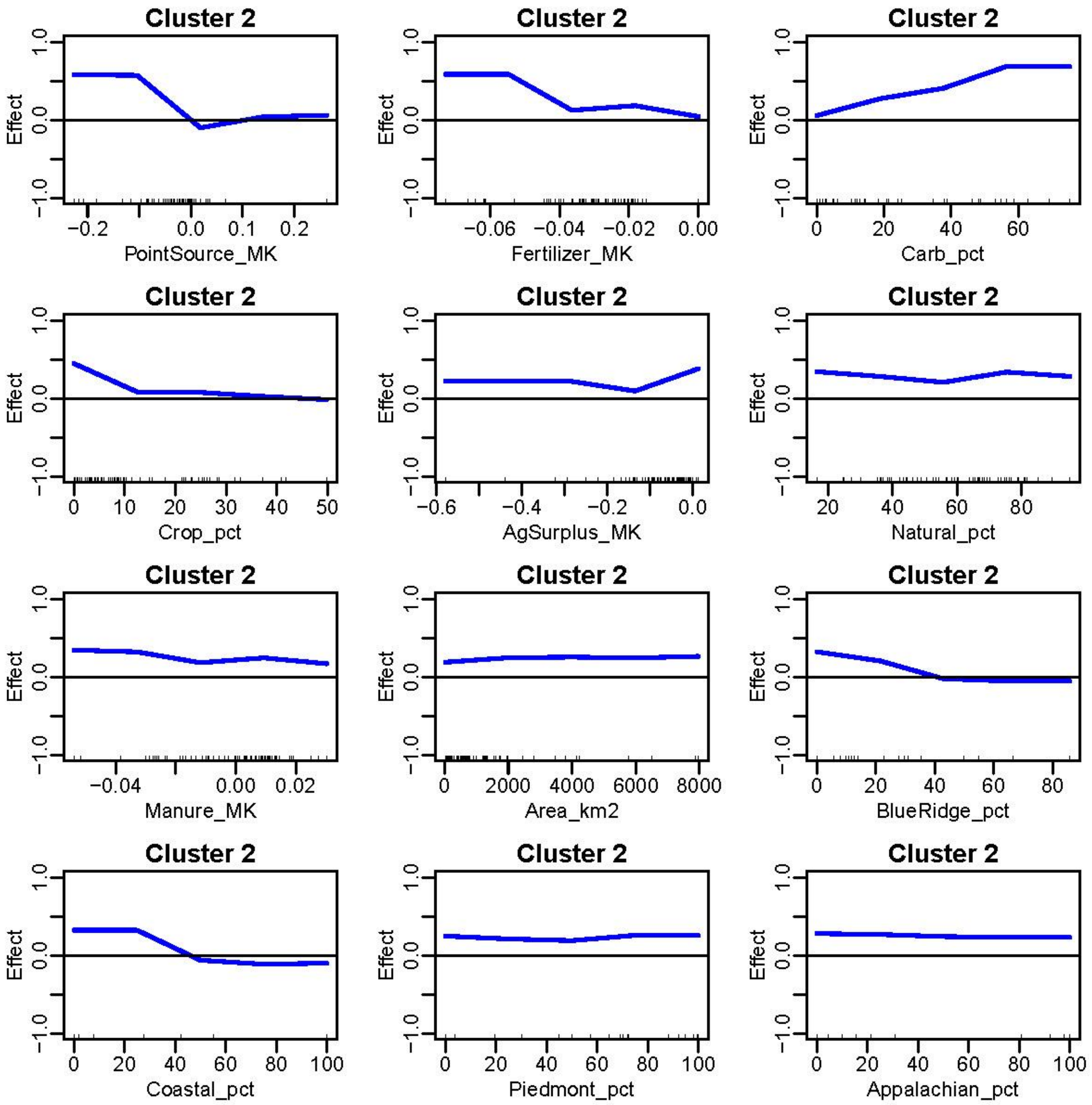

Figure S6. Partial dependence plots for all the candidate features in the base random forest model, showing their marginal effect on the probability of cluster 2.

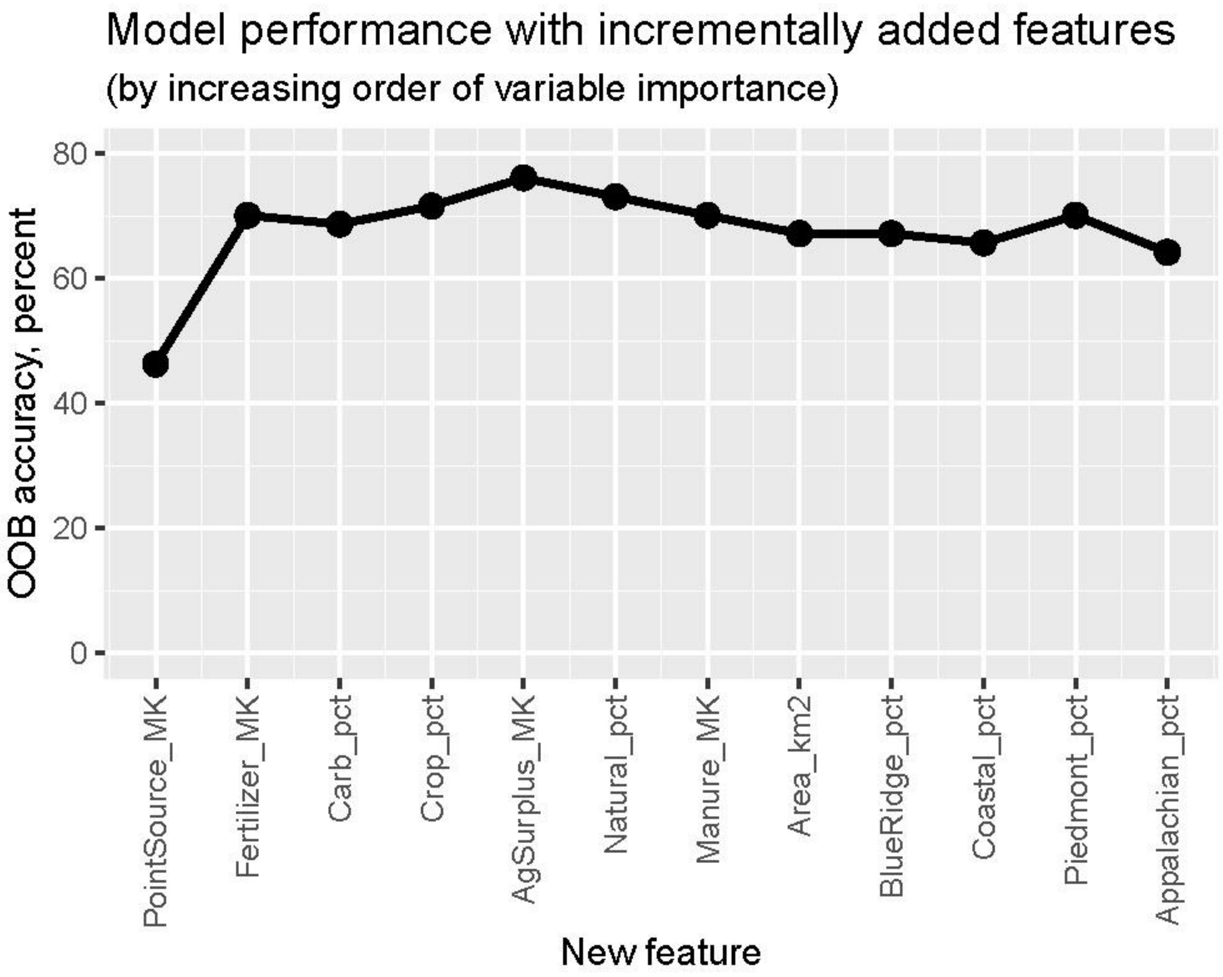

Figure S7. Experimentation showing that the random forest model accuracy reached a plateau at about five features and additional features failed to improve the model accuracy. Y-axis shows the out-of-bag (OOB) accuracy. X-axis shows the features that were incrementally added to the model. The first data point indicates the model with the first feature on the x-axis (i.e., PointSource_MK), the nth data point indicates the model with the first n features on the x-axis, and the last data point indicates the model with all the features on the x-axis. The order of variable importance was determined in the base model (Figure S5a).

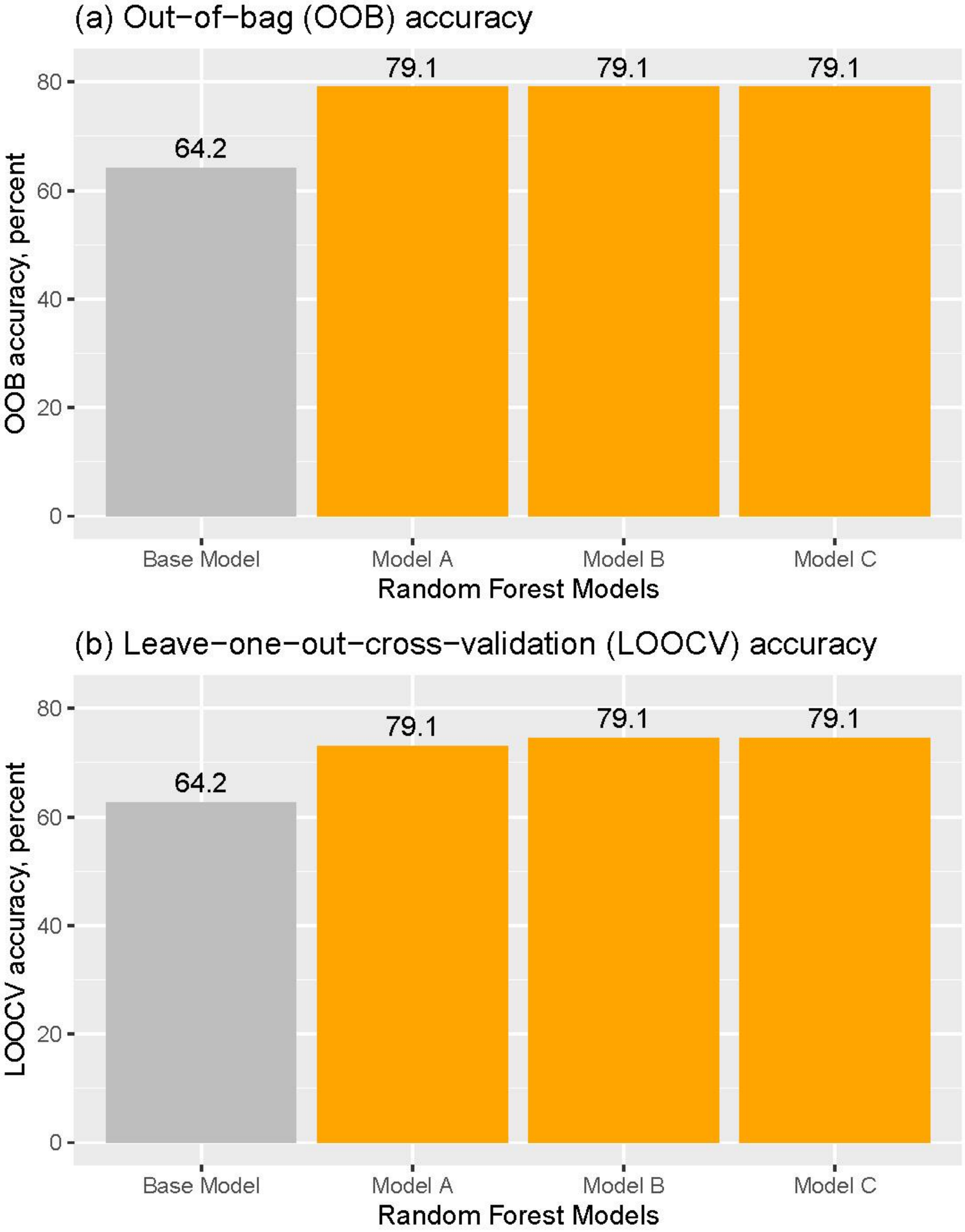

Figure S8. Barplots showing (a) the out-of-bag (OOB) accuracies and (b) the leave-one-out-cross validation (LOOCV) accuracies for the base random forest model and the three optimal models.

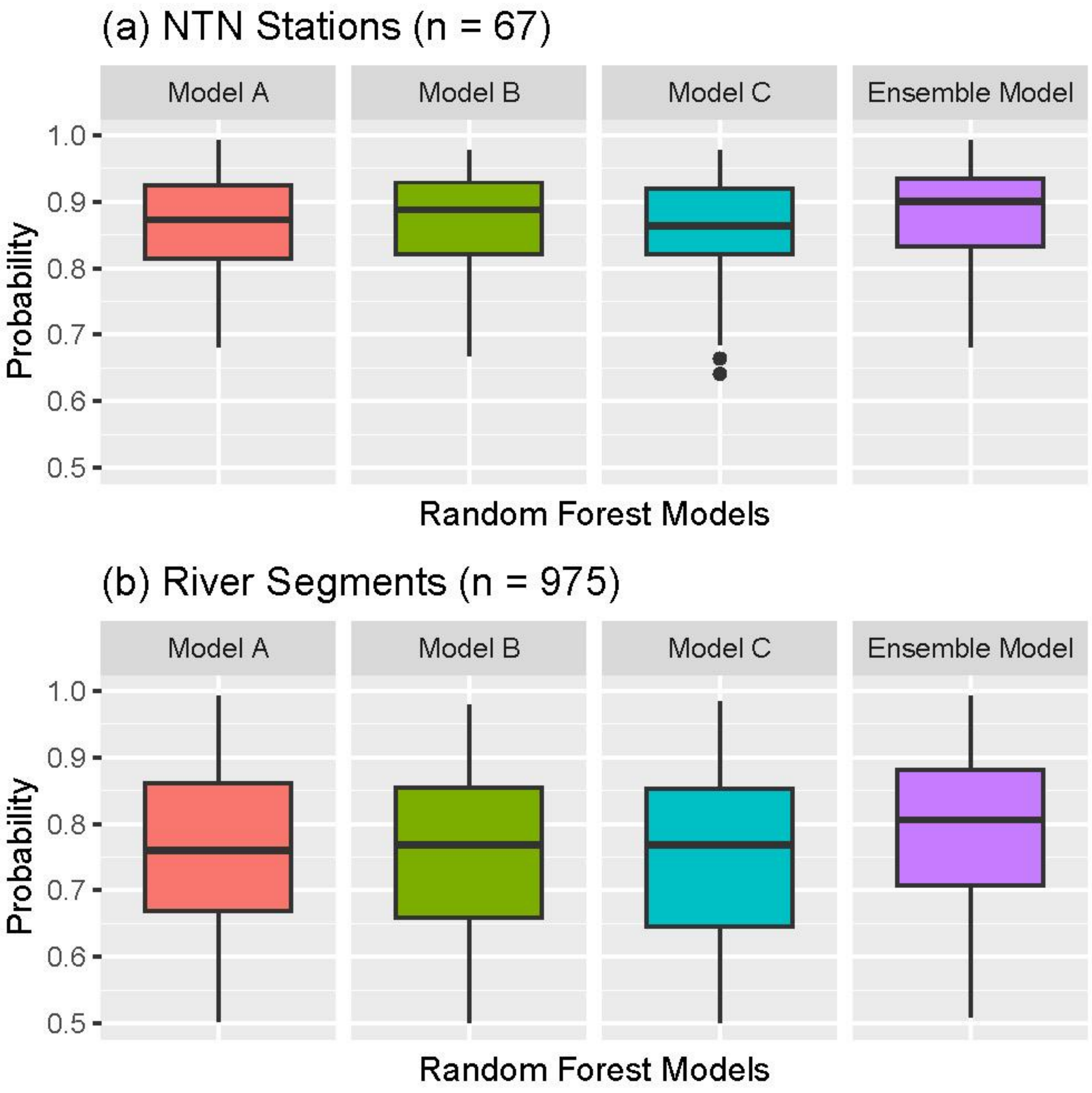

Figure S9. Boxplots showing the probability associated with each prediction, as reported by the random forest models for (a) the NTN stations and (b) the river segments. The ensemble model approach always selected the highest probability from the three optimal models.

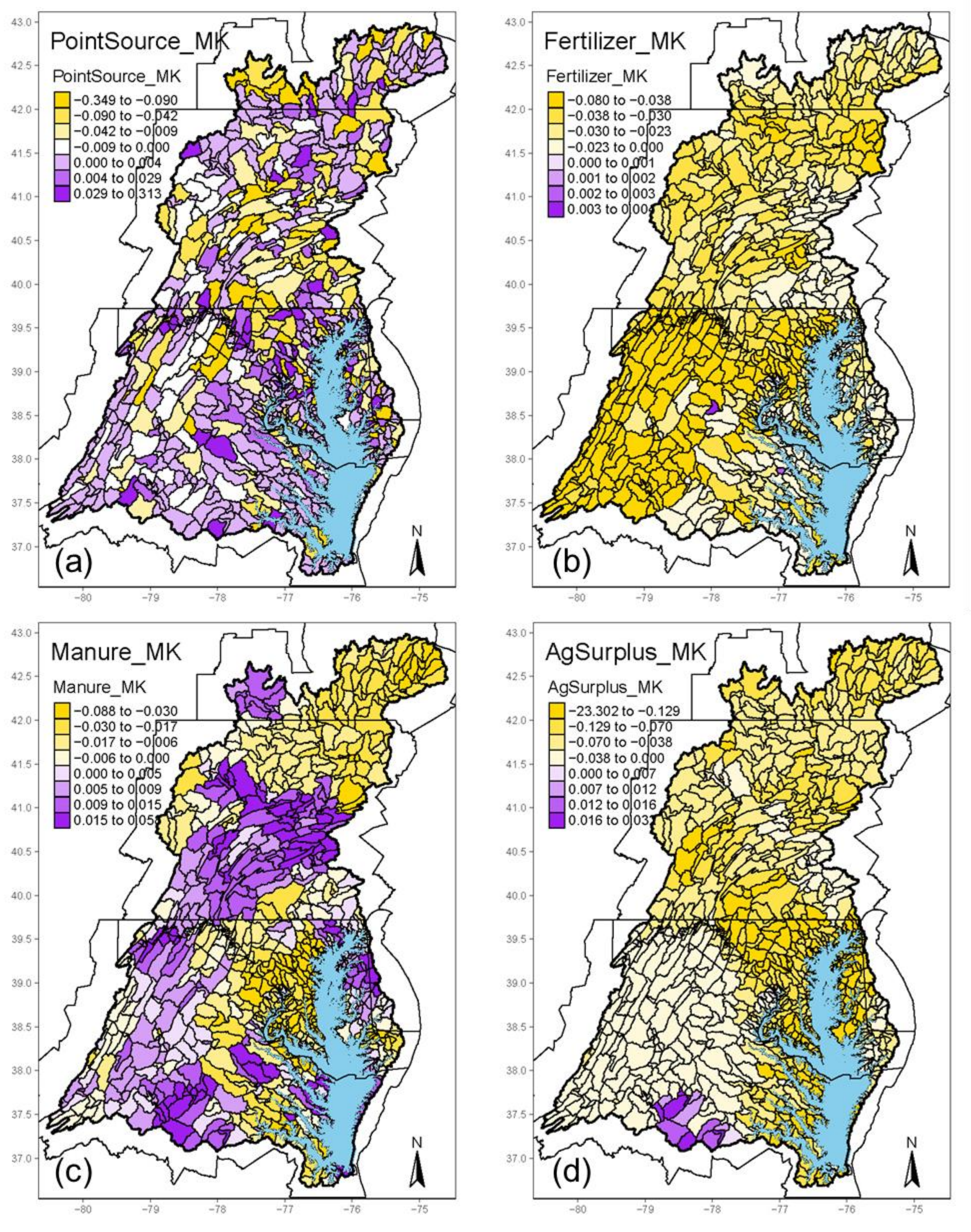

Figure S10. Map of the source-related features at the scale of river segments: (a) PointSource_MK, (b) Fertilizer_MK, (c) Manure_MK, and (d) AgSurplus_MK. See Table 1 for feature definitions.

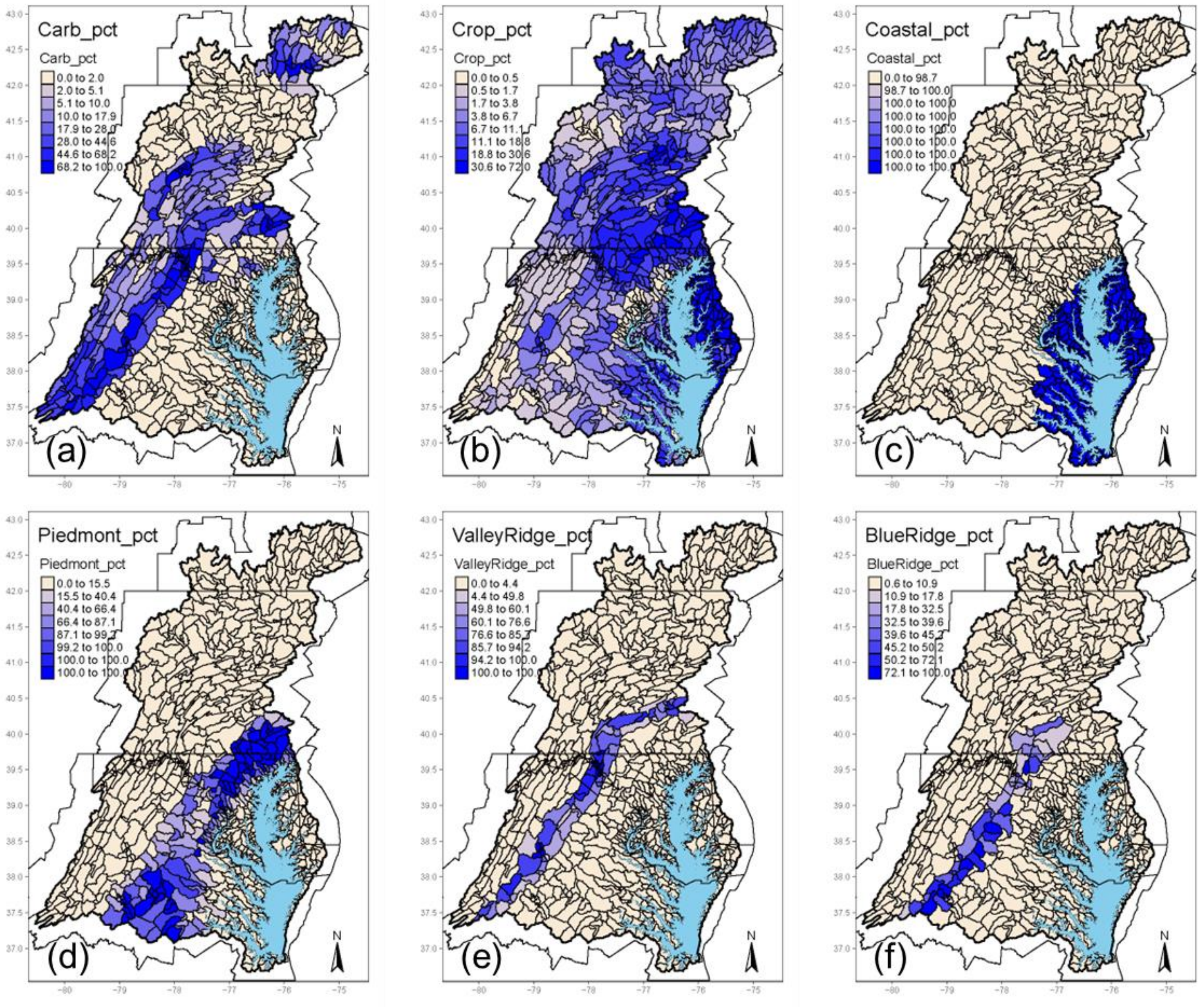

Figure S11. Map of the non-source-related features at the scale of river segments: (a) Carb_pct, (b) Crop_pct, (c) Coastal_pct, (d) Piedmont_pct, (e) ValleyRidge_pct, and (f) BlueRidge_pct. See Table 1 for feature definitions.

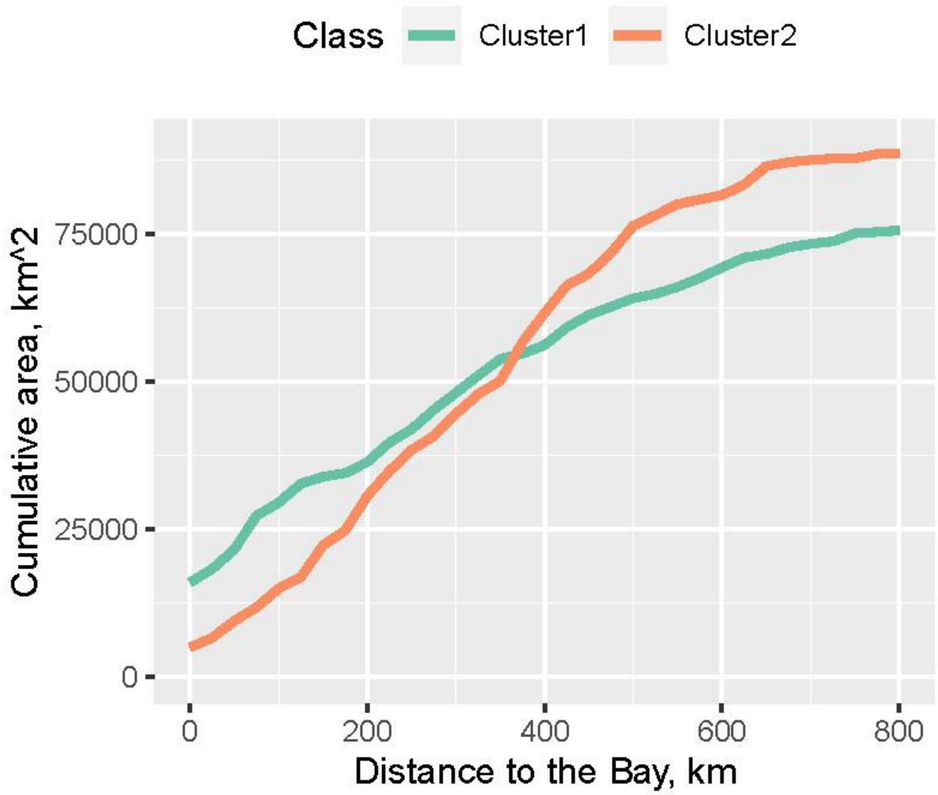

Figure S12. Cumulative area of the river segments for each cluster as a function of distance to the Bay.
